# DNA binding residues in the RQC domain of Werner protein are critical for its catalytic activities

**DOI:** 10.18632/aging.100463

**Published:** 2012-06-13

**Authors:** Takashi Tadokoro, Tomasz Kulikowicz, Lale Dawut, Deborah L. Croteau, Vilhelm A. Bohr

**Affiliations:** Laboratory of Molecular Gerontology, National Institute on Aging, Baltimore, MD 21224, USA

**Keywords:** RecQ helicase, WRN, RQC domain, WH-motif, DNA unwinding, exonuclease

## Abstract

Werner protein (WRN), member of the RecQ helicase family, is a helicase and exonuclease, and participates in multiple DNA metabolic processes including DNA replication, recombination and DNA repair. Mutations in the WRN gene cause Werner syndrome, associated with premature aging, genome instability and cancer predisposition. The RecQ C-terminal (RQC) domain of WRN, containing α2-α3 loop and β-wing motifs, is important for DNA binding and for many protein interactions. To better understand the critical functions of this domain, we generated recombinant WRN proteins (using a novel purification scheme) with mutations in Arg-993 within the α2-α3 loop of the RQC domain and in Phe-1037 of the μ-wing motif. We then studied the catalytic activities and DNA binding of these mutant proteins as well as some important functional protein interactions. The mutant proteins were defective in DNA binding and helicase activity, and interestingly, they had deficient exonuclease activity and strand annealing function. The RQC domain of WRN has not previously been implicated in exonuclease or annealing activities. The mutant proteins could not stimulate NEIL1 incision activity as did the wild type. Thus, the Arg-993 and Phe-1037 in the RQC domain play essential roles in catalytic activity, and in functional interactions mediated by WRN.

## INTRODUCTION

Werner Syndrome (WS) is a rare autosomal recessive disorder characterized by premature aging. Cells from WS patients show elevated levels of DNA deletions, translocations, chromosomal breaks, and display replicative defects, including an extended S-phase and premature senescence [1, 2]. The WRN gene, encoding Werner protein, has been identified as defective in WS. WRN belongs to the RecQ helicase family, members of which are ubiquitously conserved from bacteria to human [3], and have been implicated in various DNA metabolic pathways, including DNA replication, recombination, and DNA repair [4, 5].

**Figure 1:**
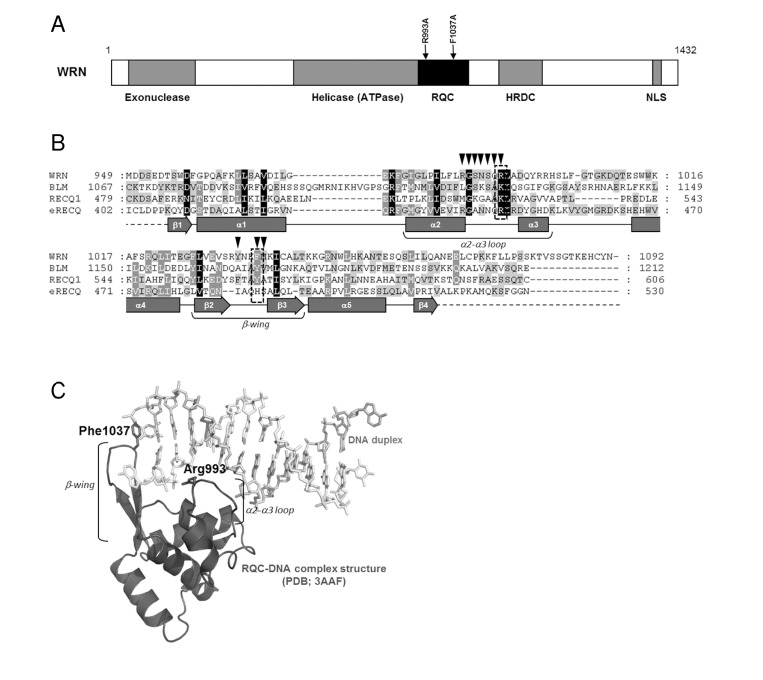
Structure and mutation sites of WRN helicase (**A**) Domain structure of WRN. Functional domains are indicated below the structure. (**B**) Amino acid sequence alignment of RQC domain of RecQ helicases. Human WRN RQC (WRN), human BLM RQC (BLM), human RECQL1 RQC (RECQ1) and E. coli RecQ RQC (eRECQ) are shown. Secondary structures of WRN RQC domain are represented below the sequences. ▼ indicates DNA-contacting amino acid residues in WRN RQC-DNA complex (PDB ID: 3AAF). Mutated sites indicated with broken line box. (**C**) Structure of WRN RQC-DNA complex (PDB ID: 3AAF). The figure generated using PyMOL (DeLano, http://pymol.sourceforge.net/).

WRN exhibits DNA-dependent ATPase, ATP dependent 3'→5' DNA helicase, single strand DNA annealing and exonuclease activities. The enzyme is able to resolve a variety of DNA substrates, including forks, flaps, displacement loops (D-loops), bubbles, Holliday junctions (HJ) and G-quadruplexes (G4), all of which represent intermediates in DNA replication and DNA repair. WRN protein consists of 1,432 amino acid residues with multiple domains, including helicase (ATPase), exonuclease, RecQ C-terminal (RQC), and helicase-and-RNaseD-like-C-terminal (HRDC) domains (Figure 1A). The major distinguishing feature of the RecQ helicase family is the RQC domain, which is composed of a zinc-binding module and a helix-turn-helix fold, a so-called winged-helix (WH) motif. The domain is important for protein function, since its deletion resulted in significant decrease in helicase activity of WRN [6] and BLM [7], and since most of the WRN protein partners interact with either entire, or at least with a part of this region [5].

A recent crystallographic study of the WRN-DNA duplex complex revealed that the β-hairpin of the WH motif binds to blunt DNA termini and un-pairs them, thereby allowing the loop between the α2 and α3 helices (α2-α3 loop) to bind within the major groove of the DNA. This suggests that the extended β-hairpin may play an important role in the DNA strand separation activity of WRN (Figure 1) [8]. A recently solved structure of a complex of the human RECQL1 with DNA also demonstrates a similar binding model (PDB ID: 2WWY). Thus, it can be suggested that the RQC domain, a unique feature of RecQ helicases, may be a key structural element regulating enzymatic activity of this class of helicases.

In this study, we have purified and characterized WRN mutant proteins with single amino acid substitutions in the RQC domain. We here present a novel purification scheme for WRN utilizing a dual tagged plasmid construct. The mutations, R993A in the α2-α3 loop and F1037A in the β-wing, were chosen based on the structure of the WRN RQC-DNA duplex complex (Figure 1). Our mutagenesis study demonstrated that these mutations cause a significant decrease, or loss of helicase activity on many tested DNA substrates. Surprisingly, the mutant proteins exhibit severe decrease in exonuclease activity, and in the single strand DNA annealing activity. The RQC domain has not previously been reported to affect these catalytic activities. We also showed that the mutant proteins are folded properly as is the wild type. These results suggest that Arg-993 and Phe-1037 in the WH motif play critical roles in catalytic activities of the WRN protein.

## RESULTS

### WRN expression and purification

For over a decade, purification of WRN helicase has been performed in our laboratory using a cumbersome and time-consuming procedure [9]. Recently, a new approach was devised, resulting in a reduction of the number of chromatographic steps, increased purity of the final product, and in overall simplification of the entire process. The new protocol uses Invitrogen's Bac-to-Bac® baculovirus expression system with a modified pFastBac1 vector. The modified vector, pFastBac1-InteinCBDAla, contains intein/chitin binding domain (InteinCBD) from the pTXB1 plasmid (IMPACT™ Kit, New England Biolabs) inserted downstream from the multiple cloning site. Additionally, SmaI restriction site and Alanine codon were incorporated in front of the InteinCBD to allow for cloning of blunt-ended inserts, and to improve efficiency of the intein self-cleavage, respectively (described in Experimental Procedures).

**Figure 2:**
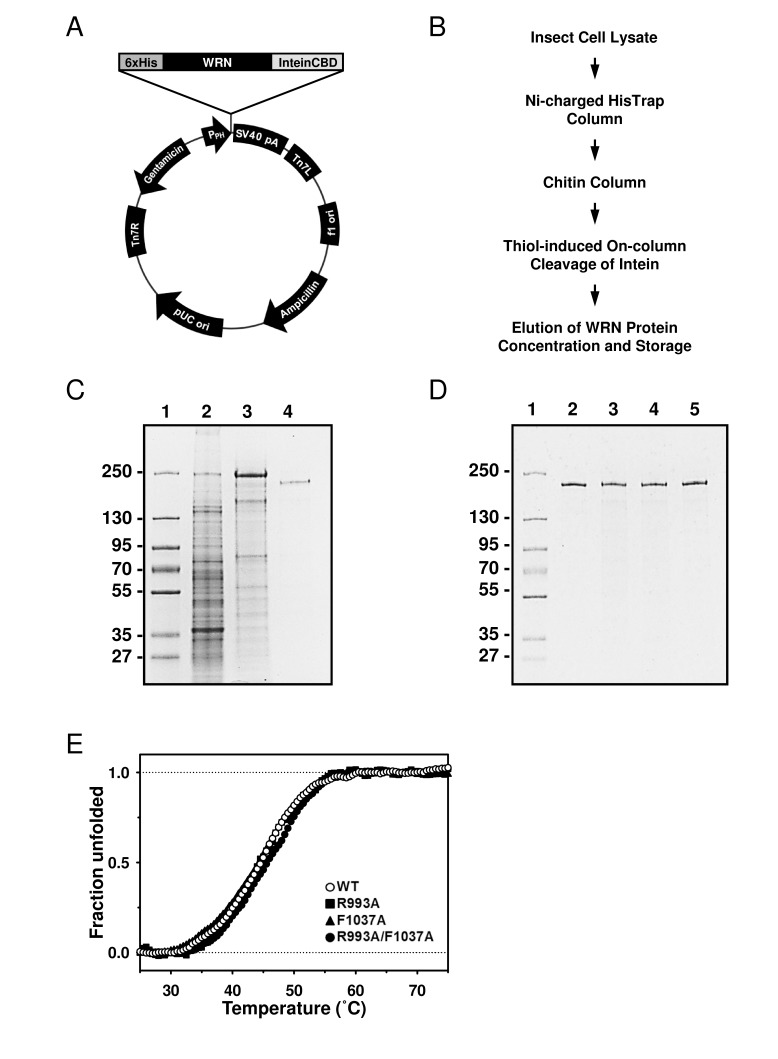
Purification of the WRN wild type and mutant proteins using new constructs (**A**) The map of 6xHis-WRN-FLAG/pFastBac1-InteinCBDAla construct, total size 9857 bp. (**B**) Improved WRN purification scheme. (**C**) SDS-PAGE showing steps of WRN purification. Lane 1, molecular marker; lane 2, whole cell extract; lane 3, pooled fractions from HisTrap column; lane 4, pooled fractions from chitin column. (**D**) SDS-PAGE of purified WRN variants. Lane 1, molecular marker; lane 2, WRN wild type; lane 3, WRN R993A; lane 4, WRN F1037A; lane 5, WRN R993A/F1037A. (**E)** Heat denaturation curves of WRN variants. 2 μg of WRN or WRN mutant was incubated with serial dilutions of SYPRO Orange. The curves were obtained by monitoring the change of fluorescence intensity given by qPCR equipment. Observed data was normalized as described in Experimental Procedures.

The WRN gene was PCR-amplified using primers incorporating hexahistidine (6xHis) and DYKDDDDK (FLAG tag) at the 5' and 3' termini, respectively. The PCR product was inserted between NotI and SmaI sites of the pFastBac1-InteinCBDAla plasmid, forming the 6xHis-WRN-FLAG/pFastBac1-InteinCBDAla construct (Figure 2A), which drives expression of WRN protein with N-terminal 6xHis and C-terminal InteinCBD tags. Next, the construct was used with the Bac-to-Bac® Baculovirus Expression System (Invitrogen) to generate recombinant baculoviruses, which were then used to express the dual-tagged WRN protein in High Five™ insect cells. The purification scheme consisted of two chromatographic steps. First, the cell lysate was applied to nickel-charged HisTrap column, followed by a chitin column. Chitin bead-bound 6xHis-WRN-FLAG-InteinCBD fusion protein was incubated overnight in the presence of thiols to induce the self-cleavage activity of the intein. Post-cleavage, the InteinCBD domain remained bound to the chitin resin, while released 6xHis-WRN-FLAG protein was eluted, concentrated and stored at -80°C, as described in Experimental Procedures (Figure 2B, 2C). The presence of two affinity tags at opposite termini simplified the purification scheme, allowed for elimination of most proteolytic fragments, and increased recovery of the full length WRN protein. The FLAG tag was not employed during purification, and was placed at the C-terminus of WRN to be utilized in future experiments.

### Effect of the mutations on DNA unwinding activity

The recently solved crystal structure of the WRN RQC-DNA duplex complex demonstrated that the amino acid residues in the α2-α3 loop and the β-hairpin motifs of the RQC domain are responsible for the DNA binding [8]. Thus, we hypothesized that the RQC domain may be important for WRN's DNA unwinding activity. For this purpose, two mutant proteins with single amino acid substitutions, R993A and F1037A, as well as a double mutant R993A/F1037A, have been isolated and biochemically characterized. The sites were chosen because mutations in Arg-993 and Phe-1037 displayed most significant reductions in DNA binding affinity [8]. First, the WRN wild type and mutant proteins were purified and their purity estimated to be > 90% by SDS-PAGE (Figure 2D). We performed heat denaturation experiment to evaluate folding of purified proteins. SYPRO Orange fluorescent dye (SO), which increases its fluorescence emission intensity upon binding with hydrophobic residues of the unfolded protein, can be used for monitoring of protein unfolding, and thereby indirectly protein structure [10, 11]. Here, we used the SO dye to monitor heat denaturation curves, and to determine the melting temperatures of the wild type and mutant WRN proteins. As shown in Figure 2E, WRN wild type, R993A, F1037A and R993A/F1037A gave similar heat denaturation profiles, suggesting that all proteins were folded similarly. The melting temperatures (Tmobs) of these proteins were around 45 °C, as summarized in Table 1, suggesting that stabilities of the mutant proteins were comparable to that of the wild type protein.

**Table 1:** The melting temperature (Tmobs) of WRN wild type and mutant proteins

Protein	Tmobs (°C)	ΔTmobs (°C)
WRN wild type	44.5	─
R993A	44.6	+ 0.1
F1037A	44.0	− 0.5
R993A/F1037A	45.4	+ 0.9

These values were obtained from heat denaturation curves of protein. The experiment carried out duplicate and errors are ±1.1 °C. ΔTmobs = Tmobs (mutant) − Tmobs (wild type).

**Figure 3:**
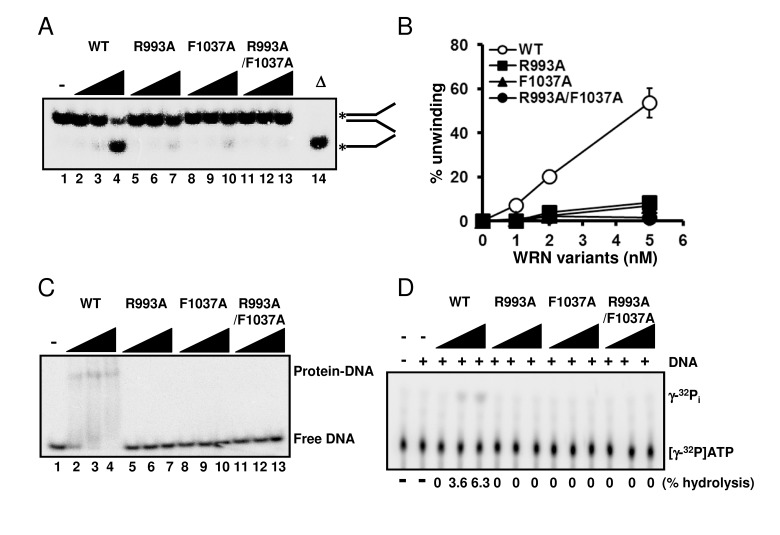
Mutations in RQC domain of WRN result in significant decrease in DNA unwinding, DNA binding and ATPase activity (**A**) DNA unwinding activity of WRN variants on forked duplex substrate. 1, 2, 5 nM WRN wild type (lane 2 to 4) or WRN RQC variants (lane 5 to 7, R993A; lane 8 to 10, F1037A; lane 11 to 13, R993A/F1037A) were incubated with 0.5 nM DNA substrate at 37 °C for 30 min. Reaction products were separated on 8% polyacrylamide gel. D indicates heat-denatured substrate control. (**B**) Quantitative analysis of (**A**). Experiments were repeated at least three times, error bars represent ± SD. (**C**) Electro mobility shift assay (EMSA) using forked duplex. 1, 2, 5 nM WRN wild type (lane 2 to 4) or WRN variants (lane 5 to 7, R993A; lane 8 to 10, F1037A; lane 11 to 13, R993A/F1037A) were incubated with 0.5 nM forked duplex substrate on ice for 30 min, then products were separated on 4 % polyacrylamide gel. (**D**) ATPase hydrolysis is disrupted by mutations. Representative polyethyleneimine thin-layer chromatography plate of ATP hydrolysis by wild type WRN or mutant proteins in the presence or in the absence of dsDNA is shown.

Since we had confirmed that the structural properties of the wild type and mutant proteins were similar, we performed helicase assays on forked duplex substrates using these recombinant proteins. All mutants, WRN R993A, WRN F1037A, and WRN R993A/F1037A, showed significantly decreased DNA unwinding activity on forked duplex (Figure 3A, 3B). It is noted that DNA unwinding by these mutants was detected when the protein concentration for the assay was increased excessively (see Supplementary Figure S1). We then examined DNA binding abilities of these mutant proteins by performing EMSA. R993A, F1037A and R993A/F1037A did not show any DNA binding affinity, similarly to the results demonstrated in a previous study where the WRN RQC domain fragment was used with the corresponding mutations (Figure 3C) [8]. We also determined whether the mutant proteins possessed ATPase activity, and found that they did not exhibit ATP hydrolysis in the presence of either ssDNA or dsDNA (Figure 3D).

**Figure 4:**
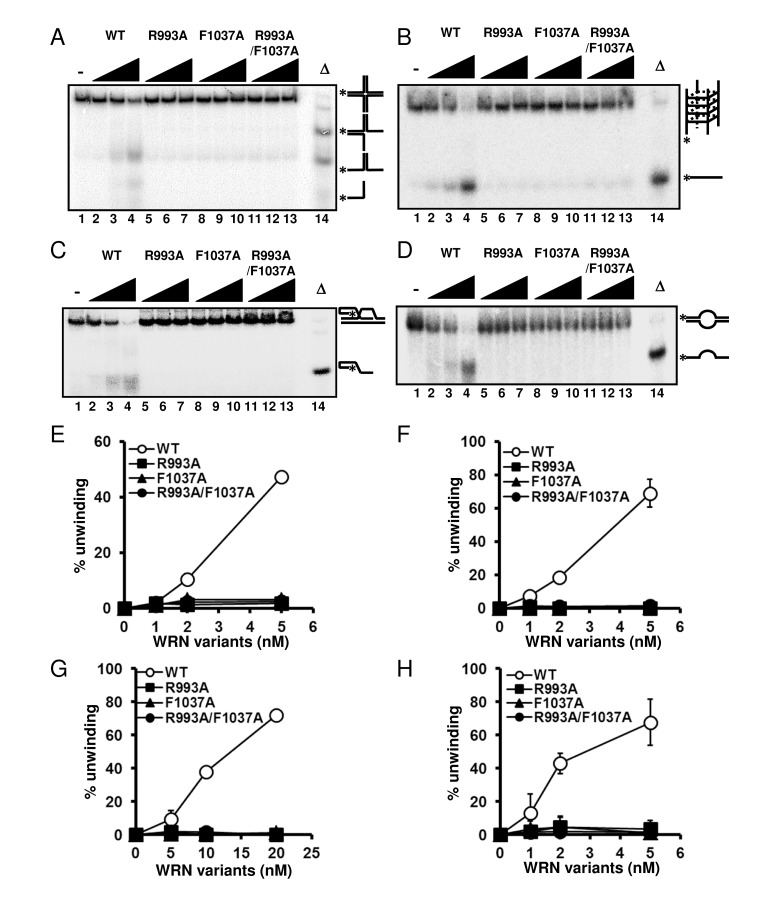
WRN RQC mutants are not able to resolve different structures of DNA substrates DNA unwinding activity on (**A**) Holliday junction (HJ) like substrate, (**B**) G-quadruplex (G4) substrate, (**C**) D-loop substrate and (D) bubble substrate. 1, 2, 5 nM WRN wild type (lane 2 to 4) or WRN mutants (lane 5 to 7, R993A; lane 8 to 10, F1037A; lane 11 to 13, R993A/F1037A) were incubated with either HJ, G4 or bubble substrate. 5, 10, 20 nM WRN wild type or WRN mutants were incubated with D-loop substrate. Reactions were performed as described in Experimental Procedures. Δ indicates heat-denatured substrate control. (**E**), (**F**), (**G**), (**H**) Quantitative analysis corresponding to (**A**), (**B**), (**C**), (**D**), respectively. Experiments were repeated at least three times, error bars represent ± SD.

WRN helicase is able to resolve a variety of DNA structures, including forks, flaps, D-loops, bubbles, HJ and G4 DNAs, all of which are thought to represent intermediates in DNA replication and recombination processes [3]. Therefore, we further examined the DNA unwinding activity of the WRN variants on HJ, G4, D-loop and bubble DNA substrates. As shown in Figure 4, WRN R993A, F1037A and R993A/F1037A could not resolve any of these DNA structures. These results strongly support the hypothesis that the β-hairpin structure and the unique DNA binding mode of RQC play integral roles in the specialized activities of WRN, and this may also be the case for other human RECQ helicases with conserved RQC structure.

### Effect of the mutations on WRN exonuclease and DNA annealing activities

**Figure 5:**
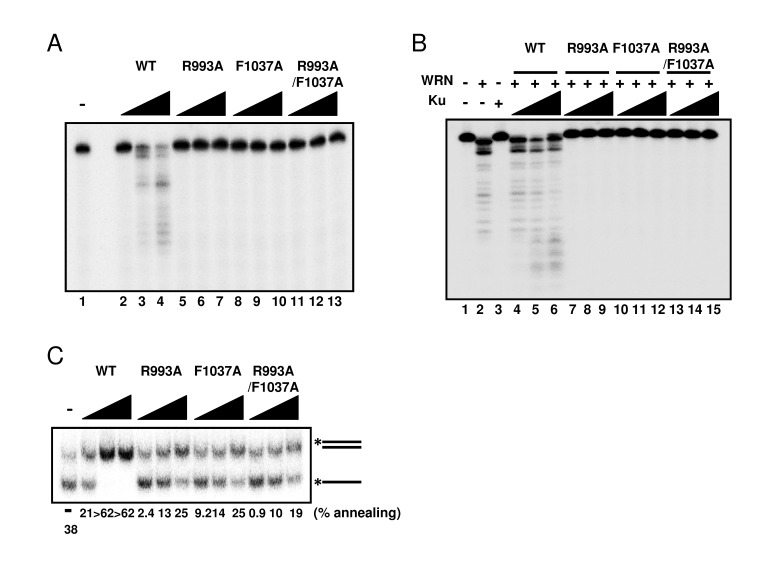
Mutations in RQC domain of WRN result in significant decrease of exonuclease activity and strand annealing activity (**A**) Exonuclease activity of WRN mutants was severely reduced. 5, 10, 20 nM WRN wild type (lane 2 to 4) or WRN variants (lane 5 to 7, R993A; lane 8 to 10, F1037A; lane 11 to 13, R993A/F1037A) were incubated with 0.5 nM 5' overhang duplex substrate. Products were separated on 14 % denaturing polyacrylamide gel. (**B**) Ku70/80 heterodimeric protein is not able to stimulate exonuclease activity of WRN mutants. 10 nM WRN wild type (lane 4 to 6) or WRN mutants (lane 7 to 9, R993A; lane 10 to 12, F1037A; lane 13 to 15, R993A/F1037A) were incubated with 5, 10, 20 nM Ku heterodimer and substrate. (**C**) WRN variants exhibit significantly lower strand annealing activity than the WRN wild type protein. 1, 2, 5 nM WRN wild type or WRN RQC variants were incubated with 0.5 nM DNA substrate at 37 °C for 15 min.

Since WRN helicase is known to exhibit other enzymatic activities, such as 3'→5' exonuclease and DNA strand annealing, we tested these activities using the WRN mutant proteins. First, we examined exonuclease activity using a 5' overhang DNA substrate. Surprisingly, neither R993A nor F1037A mutant proteins exhibited exonuclease activity (Figure 5A), suggesting that the RQC domain is not only responsible for the helicase activity, but is also involved in regulating the exonuclease activity. WRN exonuclease is known to be stimulated by Ku70/80 heterodimeric protein [12-14]. Therefore, we expected that the interaction between Ku protein and WRN mutants would stabilize the WRN mutant-DNA substrate complex structure, thereby improving their exonuclease activity. As shown in Figure 5B, however, Ku heterodimer was not able to stimulate exonuclease activity of any mutant protein (Figure 5B: lanes 7-15), while, as reported previously, it stimulated the exonuclease activity of wild type WRN (Figure 5B: lanes 4-6). We also examined the DNA strand annealing activity of the WRN mutants. As shown in Figure 5C, R993A, F1037A and R993A/F1037A exhibited significantly lower activity than the wild type (< 15% of wild type), suggesting the involvement of the RQC domain in strand annealing activity as well. We have previously demonstrated using truncated proteins that the region between the RQC and HRDC of WRN is required for ssDNA annealing [15]. However, our results in this study suggest that the RQC domain itself may also contribute to the strand annealing activity of the full length protein.

### Assessment of mutant protein folding

**Figure 6:**
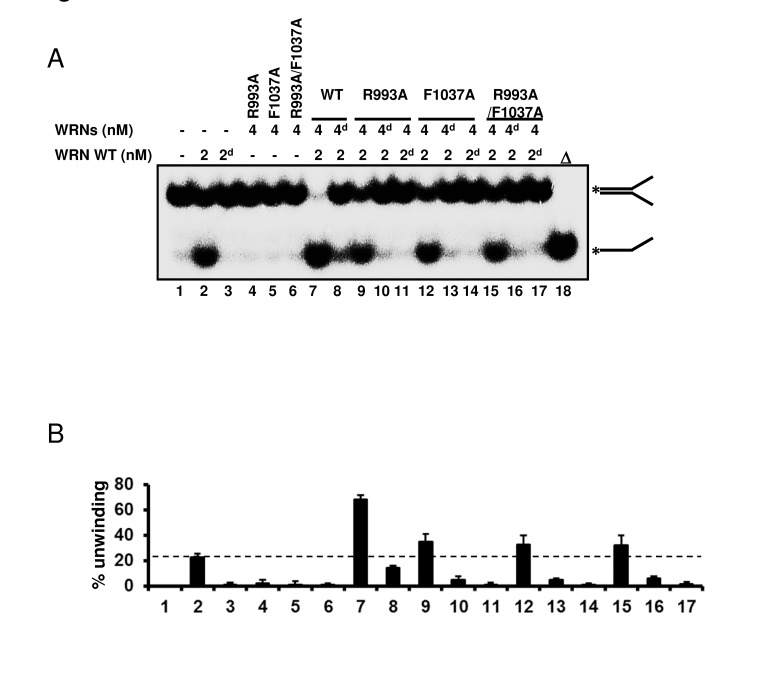
WRN RQC mutants are folded properly (**A**) WRN mutants are able to stimulate DNA unwinding activity of wild type WRN by forming hetero oligomeric structure. 2 nM of either native or heat-denatured WRN wild type (2d, heat denatured form) was mixed with 4 nM of either native or heat-denatured WRN mutant (4d, heat denatured form), and incubated with 0.5 nM forked duplex substrate at 37 °C for 20 min. Reaction products were separated on 8% polyacrylamide gel. Δ indicates heat-denatured substrate control. (**B**) Quantitative analysis of (**A**). Experiments were repeated at least three times, error bars represent ± SD.

Since our biochemical experiments showed that mutations in the RQC domain cause loss of all enzymatic activities of WRN, we then asked whether the mutant proteins were folded properly. An earlier gel filtration study suggested that WRN protein exists as a trimer form in solution [16]. However, electron microscopy studies suggested that WRN is likely to be a dimer in solution while it forms higher oligomers such as a tetramers in the presence of DNA substrate [17]. These findings led us to hypothesize that the oligomeric status of WRN protein, which changes in the presence of the DNA substrate, may be important for its enzymatic functions. Thus, if the mutant proteins were folded properly, they might form higher oligomeric structures with the wild type protein when mixed in the presence of DNA substrates. The hetero-oligomer could thereby facilitate DNA unwinding, but at a lower level of activity than the oligomer consisting of wild type only. Based on this notion, we performed helicase assays with mixed wild type and mutant proteins. The representative gel image and summarized data are shown in Figure 6. When 2 nM wild type WRN alone (lane 2) was compared with 2 nM wild type with 4 nM R993A mutant (lane 9), the helicase activity increased an additional 10 to 15 %. This is more than additive effect, suggesting that the R993A mutant protein was able to stimulate the helicase unwinding activity of WRN wild type. Similar results were observed for F1037A and R993A/F1037A (Figure 6A, 6B: lane 12, lane 15). The above results, taken together with the heat denaturation experiment (Figure 2E), indicate that mutant proteins, R993A, F1037A and R993A/F1037A are most likely folded properly.

**Figure 7:**
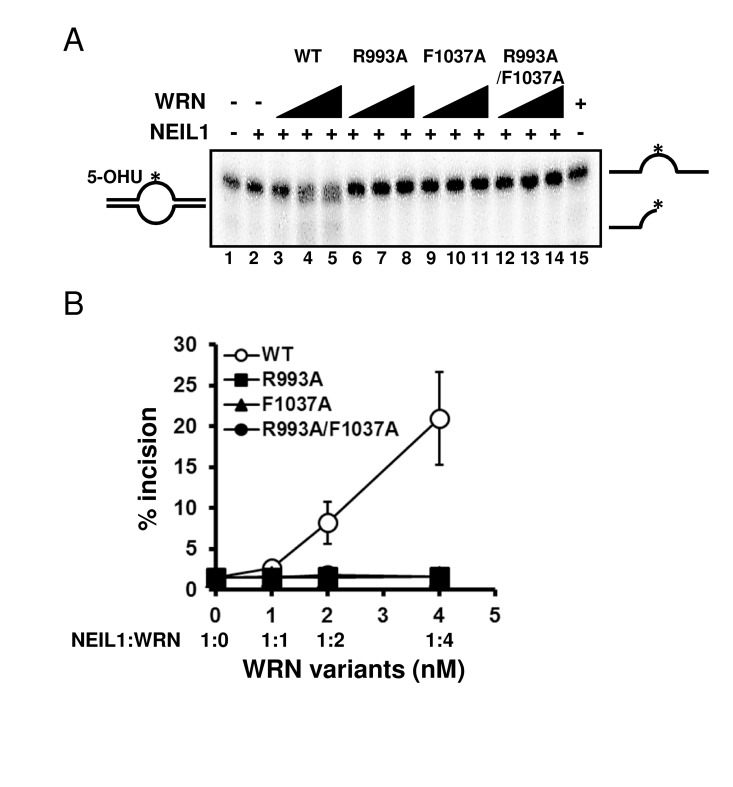
WRN RQC mutants are not able to stimulate NEIL1 incision activity (**A**) NEIL1 incision activity in the presence of WRN mutants. 1 nM of NEIL1 was incubated with 0, 1, 2 or 4 nM of WRN wild type (lane 3 to 5) or WRN variants (lane 6 to 8, R993A; lane 9 to 11, F1037A; lane 12 to 14, R993A/F1037A) in the presence of 0.5 nM of DNA substrate at 37 °C for 10 min. Reaction products were separated on 10 % denaturing polyacrylamide gel. (**B**) Quantitative analysis of (**A**). Experiments were repeated at least three times, error bars represent ± SD.

Mutations disrupt the functional interaction with NEIL1 Several studies have revealed that the RQC domain of WRN is important not only for DNA binding but also for protein-protein interactions, including FEN1 [18], NEIL1 [19], PARP1 [20], p97/VCP [21] and TRF2 [22]. We have previously reported that WRN containing a K577M mutation, which inactivates the helicase activity, retained the ability to stimulate NEIL1's incision activity [19]. Therefore we chose to investigate how the WRN mutations introduced in this study affected the functional interaction with NEIL1. We performed the NEIL1 incision assays using a 5-OHU-containing bubble substrate. The results showed that the wild type WRN stimulated NEIL1's incision activity in a concentration dependent manner (Figure 7A: lanes 3 to 5, Figure 7B), whereas the mutant proteins could not stimulate NEIL1's activity (Figure 7A: lanes 6 to 14, Figure 7B).

## DISCUSSION

A recent crystallographic study suggested that the winged-helix motif of the RQC domain could play a central role in the DNA unwinding activity of WRN [8]. The results of this study strongly support the hypothesis that WRN helicase uses the RQC WH structure for base-pair separation. The reduction in helicase activity of the mutant proteins underscores the significance of the RQC winged-helix structure residues Arg-993 and Phe-1037, which are involved in the DNA separation reaction (Figure 1C). Unlike most of the other helicases, RecQ family helicases preferentially resolve a broad type of DNA structures, including forked duplex, HJ, G4, D-loop and DNA bubbles. Our results clearly indicate that the RQC domain plays a significant role in the recognition and separation of forked duplex, HJ, G4, D-loop and bubble structures (Figure 3, 4). Taken together, the central molecular element of the DNA unwinding reaction for WRN may be common for WRN regardless of the type of DNA substrate. Similar results have been observed with Tyrosine mutation in the RQC β-hairpin of RECQL1, corresponding to the Phe-1037 residue in WRN RQC (Figure 1B), which caused a great reduction in helicase activity on forked duplex and Holliday junction [23].

WRN is unique among the RecQ helicases because it possesses an N-terminal exonuclease domain, which can operate independently of the helicase activity [24, 25]. Surprisingly, we found that WRN R993A, F1037A or R993A/F1037A mutants exhibited no exonuclease activity (Figure 5A). It was previously shown that the N-terminal exonuclease can operate independently of the helicase activity [32, 38]. The isolated exonuclease domain of WRN without the RQC domain, and a Walker A motif WRN mutant, which lacks helicase activity, possess proficient exonuclease activity [24, 25], and our mutagenesis results therefore suggest that the RQC domain plays a significant role in WRN exonuclease function. We and another group have shown that full-length WRN has higher specific activity of exonuclease than a fragment of WRN containing only the exonuclease domain. A higher molar concentration of the WRN exonuclease domain alone (5 to 10 fold more) is required to achieve the level of activity equivalent to that of the full-length protein [13, 25]. It has been also shown that a unique amino-terminal region of WRN, outside the exonuclease domain, is necessary for oligomerization and increased nuclease processivity [26]. Thus, the RQC domain may contribute to the recognition and stabilization of the WRN-DNA complex and to the processivity of WRN exonuclease. It has been reported that mutations in amino acids making contact with DNA, but not in the catalytic residues, cause significant reduction in the nuclease activity of several nuclease family enzymes [27-29]. We also showed that the exonuclease activity of the mutant WRN proteins could not be stimulated by Ku protein (Figure 5B). This may be because Ku does not directly interact with the WRN RQC domain, but with its exonuclease domain and the C-terminal regions of WRN as demonstrated by domain mapping [12-14]. Since the RQC domain of WRN is not directly modulated by Ku, the exonuclease activity of WRN mutants are not enhanced by Ku. Moreover, interaction with Ku may not be able to modulate the RQC-DNA interaction. This notion is supported by the fact that Ku interaction does not influence the WRN helicase activity, which is operated mainly by the RQC domain [12].

The strand annealing activity is also deficient in the mutant WRN proteins, less than 40 % of the wild type, even though the RQC domain has not been previously implicated in this activity (Figure 5C) [15]. Thus, the RQC domain also contributes to single-stranded DNA annealing, possibly by mediating WRN-DNA structure and/or by increasing processivity of WRN. Together, our results clearly indicate that the RQC domain is not only required for helicase activity, but is also necessary for the other enzymatic activities of WRN (e. g. exonuclease activity, strand annealing activity).

We also showed that WRN RQC mutant proteins could not stimulate NEIL1's incision activity (Figure 7). We have previously demonstrated that the RQC domain of WRN interacts with NEIL1, and that this domain of WRN is sufficient to stimulate the incision of an oxidatively damaged DNA base by NEIL1 in an ATP-independent manner [19]. This suggests that proper DNA binding of WRN may affect the catalytic activity of NEIL1. Similar results were observed previously for FEN1 stimulation by WRN using a fragment with a RQC mutation [6].

WRN is mainly located in the nucleoli. We have previously demonstrated that the RQC domain is required for WRN's nucleolar localization [30]. We have also shown that WRN redistributes from the nucleolus to the nucleoplasm in response to DNA damage or replication stress, suggesting that the RQC domain is required for proper nuclear distribution of WRN [30, 31]. Further, some experiments have suggested that the nucleolus regulates cellular senescence [32, 33]. Although the role of WRN in the nucleolus is still unclear, we have reported a functional interaction between WRN and the abundant protein nucleolin which has central functions in the nucleolus [34], and this association may be an important part of WRN function and its role in limiting cellular senescence. No mutations in the RQC domain have yet been identified and this may testify to the importance of a functional WRN RQC.

The structure of the human RECQL1-DNA (PDB ID: 2WWY) complex revealed a similar binding mode as the one observed in the solved structure of WRN-WH bound to dsDNA. Despite the poor sequence similarity between the RQC domains in WRN and RECQ1, the three dimensional structures of the WH motifs are similar. Based on the amino acid sequence alignment of the RQC domains (Figure 1B), the RQC domain of BLM may have similar folds as those of RECQL1 and WRN. Moreover, the negatively charged residues in the α2-α3 loop (Arg for WRN, Lys for RECQL1, BLM) and the aromatic residue in the β-wing (Phe for WRN, Tyr for RECQL1, BLM) are well conserved. Thus, it can be suggested that the RQC domain, and especially the WH motif, is a key structural component in the regulation of the enzymatic activity of this family of helicases. In contrast, RECQL4 lacks the entire RQC sequence and RECQL5 has an incomplete RQC domain, containing the zinc-binding motif, but lacking the WH motif sequence. Interestingly, both RECQL4 and RECQL5 exhibit weak helicase activity in vitro, when compared to WRN, BLM or RECQL1. Thus, the RQC domain, and especially the WH motif, may play a central regulatory role in the efficiency of DNA separation reaction of the RecQ helicases. However, contrary to this idea, recent results from a study with purified fragments of BLM have shown that the WH motif of BLM is not required for DNA separation activity [35]. Similarly, a mutation at the corresponding position of E. coli RecQ (Histidine 497) did not affect DNA unwinding [23]. Therefore, the structural elements required for DNA separation may be different between BLM and WRN proteins. Additionally, a recent in vitro study has shown that WRN differs from BLM in DNA binding characteristics, suggesting dissimilarities in DNA binding mode and/or in DNA-binding structural element [36].

Several mutations, including insertion/deletion, missense and nonsense mutations, have so far been identified in the WRN gene derived from WS patients. The information about these mutations is well summarized and available through the International Registry of WS (http://www.wernersyndrome.org). The missense mutations (and polymorphisms) are distributed throughout the sequence [37, 38]. However, none of the missense mutations were observed within the RQC domain. A couple of nonsense mutations were found within RQC domain and in the helicase domain, which is located upstream of the RQC domain. Therefore, the relationship between the contribution of the RQC domain to the enzymatic activity of WRN and the patient phenotypes still remains unclear. Further studies are required to clarify the genotype-phenotype correlation for the WRN mutations.

## METHODS

### Plasmid construction

pFastBac1 plasmid (Invitrogen) was PCR-linearized using primers: pFastBac1-F, 5'-GTCGAGAAGTACTAGAGGATC-3', and pFastBac1-R2, 5'-GGAATTAAGCTTGGTAC CGCATG-3'. The PCR product's 5' blunt end was phosphorylated using T4 Polynucleotide Kinase (PNK, New England Biolabs), and the 3' end digested with HindIII (New England Biolabs). Mycobacterium xenopi GyrA intein/chitin binding domain fusion was PCR-amplified from pTXB1 plasmid (New England Biolabs) with primers: InteinCBD-SmaI-Ala-F, 5'-GGAATTAAGCTTCCCGGGGCCTGCATCACGGGAGATGCAC-3', and InteinCBD-Stop-R, 5'-TCATTG AAGCTGCCACAAGGCA-3', which incorporated SmaI restriction site (underlined) and Alanine codon (italicized). The PCR product was digested with HindIII, phosphorylated using PNK and ligated into pFastBac1, resulting in the formation of pFastBac1-InteinCBDAla plasmid.

WRN gene was PCR amplified from YEp195SpGAL-WRN plasmid [39] using primers: hWRN-NotI-6xHis-F, 5'-GGAATTGCGGCCGCATGTCTCATCACCATC ACCATCACAGTGAAAAAAAATTGGAAACAAC-3' and WRN-FLAG-blns-R, 5'-CTTATCGTCGTCATC CTTGTAATCACTAAAAAGACCTCCCCTTTTC-3', adding sequences coding hexahistidine (6xHis tag), and DYKDDDDK (FLAG tag) to the 5' and 3' termini, respectively. The PCR product was digested with NotI, phosphorylated with PNK, and ligated into NotI/SmaI digested pFastBac1-InteinCBDAla vector, producing 6xHis-WRN-FLAG/pFastBac1-InteinCBDAla construct. The sequence of final construct was confirmed by direct sequencing. All DNA oligonucleotides were purchased form Integrated DNA Technologies (Coralville, IA).

Arginine 993 and Phenylalanine 1037 were substituted into Alanine by site-directed mutagenesis. The mutagenic primers were designed such that the codons for Arg (CGT) and Phe (TTT) are changed to those for Ala (GCG, GCT), respectively. We also constructed the double mutants, R993A/F0137A. The nucleotide sequences were confirmed by sequencing.

### Generation of recombinant baculoviruses

Recombinant baculoviruses were generated using Bac-to-Bac® Baculovirus Expression System (Invitrogen). Briefly, 6xHis-WRN-FLAG/pFastBac1-InteinCBDAla construct was transformed into DH10Bac™ E. coli strain (Invitrogen), and recombinant bacmids purified from large white colonies. The insertion of the 6xHis-WRN-FLAG/InteinCBDAla gene was verified by PCR. Next, bacmids were transfected into Sf9 insect cells using Cellfectin II® reagent (Invitrogen). The recombinant viruses were subsequently amplified in Sf9 cells grown at 27 °C in TNM-FH medium (Mediatech) supplemented with 10% fetal bovine serum (Sigma-Aldrich).

### Protein expression and purification

High Five™ insect cells (Invitrogen) were maintained in suspension with aeration in ESF 921 medium (Expression Systems, Woodland, CA) at 27 °C. The cells were infected with recombinant baculovirus at multiplicity of infection (MOI) of 10, grown for 48 hours and harvested by centrifugation. All purification steps were carried out at 4 °C. Insect cell pellets were sonicated in lysis buffer (50 mM Tris HCl, pH 8.0, 300 mM NaCl, 10% glycerol, 25 mM Imidazole, 1 mM PMSF, 1 tablet/50 ml Complete EDTA-free Protease Inhibitor Cocktail, Roche), spun 30 min. at 37,000g, and filtered through 45 μm membrane. Clarified lysate was loaded onto HisTrap FF 5 ml column equilibrated in Buffer A (50 mM Tris HCl, pH 8.0, 300 mM NaCl, 10% glycerol) supplemented with 25 mM Imidazole, using ÄKTApurifier instrument (GE Healthcare). The column was washed with 10 column volumes (CV) of Buffer A + 25 mM Imidazole, and the protein eluted with 10 CV of Buffer A + 250 mM Imidazole. Peak fractions containing protein of interest were pooled, and loaded 3 times by gravity flow onto 3 mL chitin column (New England Biolabs) equilibrated with Buffer A. The column was washed with additional 20 CV of Buffer A, followed by 3 CV of Buffer A + 100 mM 2-Mercaptoethanol. The flow was stopped and the intein self-cleavage reaction allowed to proceed for 18 hrs. at 4°C. Cleaved protein was eluted with 5 CV of Buffer A + 100 mM 2-Mercaptoethanol, peak fractions were pooled and concentrated using Amicon® Ultra-4 centrifugal filter unit with 50 kDa cutoff (Millipore). Purified concentrated protein was diluted 1:1 with cold 100% glycerol, aliquoted and stored at -80 °C. Protein concentration was determined by Bradford method (Bio-rad), and protein purity was analyzed on SDS-PAGE. Purifications of all three mutant proteins were performed as described above for wild type protein.

Recombinant protein of Ku70/80 heterodimer was purified as described previously [12]. Recombinant NEIL1 protein was obtained previously [40].

### Heat denaturation analysis

To determine the protein stability, thermofluor assay was carried out using an iQ cycler instrument (Bio-rad). Protein stability measurements were performed in buffer containing 50 mM HEPES-NaOH, pH 8.0, 60 mM NaCl, 1 mM DTT and 15 % (v/v) glycerol. The protein was used at 0.1 μg/μl concentration (2 μg in 20 μl reaction) and SYPRO Orange (Invitrogen) was used at 1x to 20x concentration range (1:250 to 1:5000 dilution of 5000x stock). Heat denaturing curves were observed within the temperature range of 20 °C to 90 °C, with approximately 2 °C/min rate of temperature change, collecting data every 0.5 °C. Obtained data was normalized by the van't Hoffs analysis [41].

### Helicase assay

Helicase unwinding assays were performed as described previously [42]. Reactions (20 μl) contained 0.5 nM substrate, 2.5 mM ATP and the indicated concentrations of WRN wild type and mutants in 40 mM Tris-HCl pH 8.0, 50 mM NaCl, 5 mM MgCl2, 100 μg/ml BSA, 2 mM ATP, 1 mM DTT. Reactions were carried out at 37 °C for 30 min, and terminated by the addition of 10 μl of SDS stop solution (2% SDS, 50 mM EDTA, 30% glycerol, 0.1% bromophenol blue, 0.1% xylene cyanol). Products were separated on 8% non-denaturing polyacrylamide gel. Radiolabeled DNA was visualized using Typhoon phosphorimager, (Typhoon 9400, GE Healthcare) and quantified using ImageQuant software (Molecular Dynamics). The D-loops were prepared and characterized as described previously [43]. G4 DNA substrate was prepared and helicase reaction was performed as described previously [44, 45].

### Substrate binding analysis

Electro mobility shift assays (EMSA) were performed to analyze the binding ability as described previously [46]. Binding reactions (20 μl) were conducted in standard binding buffer (40 mM Tris-HCl, pH 8.0, 1 mM EDTA, 20 mM NaCl, 8% glycerol, and 20 μg/ml BSA). Protein and DNA substrate concentrations are indicated in Figure legends. Reactions were incubated for 30 min on ice and directly loaded onto 5% non-denaturing polyacrylamide gels. Products were visualized using Typhoon phosphorImager (GE Healthcare) and quantified using ImageQuant software (Molecular Dynamics).

### ATPase assay

ATPase assays were performed as described previously [4]. Buffers for standard ATPase reactions contained 20 mM HEPES-NaOH, pH 8.0, 0.05 mM ATP, 40 μg/ml BSA, 1 mM DTT. ATPase reactions employed WRN and 12.5 μCi of [γ-32P]ATP. Reactions were incubated for 1 h at 30 °C with 150 ng of nucleic acid substrates (M13 mp18 ssDNA for ssDNA and pUC19 for dsDNA), and stopped by the addition of 5 μl of 0.5 M EDTA. ATP hydrolysis was analyzed by polyethyleneimine thin-layer chromatography using 1 M formic acid, 0.8 M LiCl mobile phase. The results were analyzed using Typhoon phosphorimager (GE Healthcare) and ImageQuant software (Molecular Dynamics).

### Exonuclease assay

Exonuclease assays were performed as described previously [12, 14]. Reactions (10 μl) were performed in buffer (40 mM Tris-HCl, pH 8.0, 4 mM MgCl2, 5 mM DTT, 2 mM ATP, and 0.1 mg/ml BSA) containing DNA substrate (0.5 nM) and WRN and/or Ku70/80 as indicated. Samples were incubated at 37 °C for 15 min. Reactions were terminated by addition of equal volume of formamide stop dye (80% formamide, 0.5×TBE, 0.1% bromophenol blue, and 0.1% xylene cyanol). Products were heat-denatured for 5 min at 95 °C, loaded on 14% denaturing polyacrylamide gels, visualized using Typhoon phosphorImager (GE Healthcare), and quantified using ImageQuant software (Molecular Dynamics).

### Single strand DNA annealing assay

DNA strand annealing assays were performed as described previously [15, 47]. C80 and G80 oligonucleotides were used. Reactions (20 μL) were carried out with 0.5 nM of each oligonucleotide, one of which was 5'-32P-end-labeled, in 20 mM Tris-HCl, pH 7.5, 2 mM MgCl2, 40 μg/mL BSA and 1 mM DTT at 37 °C for 15 min. Concentrations of WRN proteins used in the reactions were indicated in Figure legends. Reactions were stopped by the addition of stop buffer (50 mM EDTA, 1% SDS and 50% glycerol) and immediately loaded onto 8 % polyacrylamide gel. Radiolabeled DNA was detected using Typhoon Imager (GE Healthcare), and percentage of annealed oligonucleotide was quantified using ImageQuant software (Molecular Dynamics).

### NEIL1 incision assay

NEIL1 incision assays were performed as described previously [19, 40]. NEIL1 substrates used were 51-mer oligonucleotides (purchased from Midland Co.) with the damaged base (5-OHU) at residue 26. The damage-containing oligonucleotide was labeled with [γ-32P]ATP and T4 polynucleotide kinase (New England Biolabs), and annealed to the complementary oligonucleotide containing a C opposite the lesion. DNA strand cleavage at the site of the lesion due to the intrinsic AP-lyase activity of NEIL1 after base excision was assayed by incubation of 32P-labeled substrate with NEIL1 (1 nM) alone or together with WRN wild type or mutant proteins (1-4 nM) as indicated. Reactions (10 μl) were performed in a buffer containing (40 mM Tris, pH 8.0, 4 mM MgCl2, 5 mM DTT, 0.1 μg/μl BSA) at 37 °C for 15 min. After stopping the reaction with 70% formamide/30 mM NaOH, the products were analyzed by denaturing gel electrophoresis. Radiolabeled products were visualized using Typhoon phosphorImager (GE Healthcare) and quantified using ImageQuant software (Molecular Dynamics).

## SUPPLEMENTAL FIGURE

Supplementary Figure S1 Mutant proteins exhibit significantly lower helicase activity than wild type. 50, 100, 200 nM WRN wild type or WRN RQC variants were incubated with 0.5 nM DNA substrate at 37 °C for 30 min. Reaction products were separated on 8% polyacrylamide gel.
