# Supplementary figures and images for: DNA binding residues in the RQC domain of Werner protein are critical for its catalytic activities
Source: Aging (Albany NY). 2012 Jun 13;4(6):417–29. doi: 10.18632/aging.100463 (PMC3409678; doi:10.18632/aging.100463)

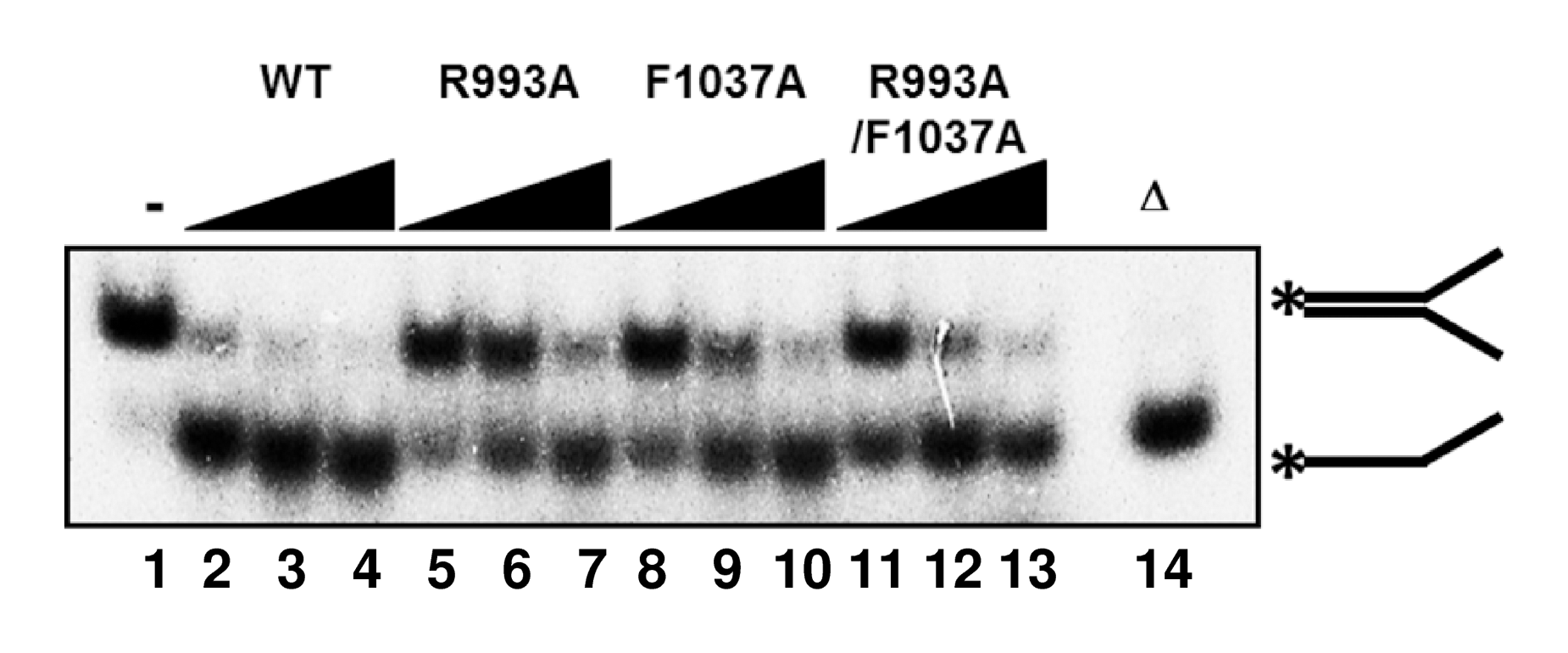

Supplement: Supplementary Figure S1 — Mutant proteins exhibit significantly lower helicase activity than wild type. 50, 100, 200 nM WRN wild type or WRN RQC variants were incubated with 0.5 nM DNA substrate at 37 °C for 30 min. Reaction products were separated on 8% polyacrylamide gel. [file aging-04-417-s001.tif]
